# Neutron Radiography Based Visualization and Profiling of Water Uptake in (Un)cracked and Autonomously Healed Cementitious Materials

**DOI:** 10.3390/ma9050311

**Published:** 2016-04-26

**Authors:** Philip Van den Heede, Bjorn Van Belleghem, Natalia Alderete, Kim Van Tittelboom, Nele De Belie

**Affiliations:** 1 Magnel Laboratory for Concrete Research, Department of Structural Engineering, Faculty of Engineering and Architecture, Ghent University, Technologiepark Zwijnaarde 904, Ghent B-9052, Belgium; philip.vandenheede@ugent.be (P.V.d.H.); bjorn.vanbelleghem@ugent.be (B.V.B.); nataliamariel.alderete@ugent.be (N.A.); kim.vantittelboom@ugent.be (K.V.T.); 2 Strategic Initiative Materials (SIM vzw), project ISHECO within the program ‘SHE’, Technologiepark Zwijnaarde 935, Ghent B-9052, Belgium; 3 LEMIT, Laboratory for Multidisciplinary Training in Technological Research, 52 entre 121 y 122 s/n, La Plata 1900, Argentina

**Keywords:** neutron radiography, capillary water absorption, mortar, cracks, autonomous self-healing, encapsulated polyurethane, viscosity

## Abstract

Given their low tensile strength, cement-based materials are very susceptible to cracking. These cracks serve as preferential pathways for corrosion inducing substances. For large concrete infrastructure works, currently available time-consuming manual repair techniques are not always an option. Often, one simply cannot reach the damaged areas and when making those areas accessible anyway (e.g., by redirecting traffic), the economic impacts involved would be enormous. Under those circumstances, it might be useful to have concrete with an embedded autonomous healing mechanism. In this paper, the effectiveness of incorporating encapsulated high and low viscosity polyurethane-based healing agents to ensure (multiple) crack healing has been investigated by means of capillary absorption tests on mortar while monitoring the time-dependent water ingress with neutron radiography. Overall visual interpretation and water front/sample cross-section area ratios as well as water profiles representing the area around the crack and their integrals do not show a preference for the high or low viscosity healing agent. Another observation is that in presence of two cracks, only one is properly healed, especially when using the latter healing agent. Exposure to water immediately after release of the healing agent stimulates the foaming reaction of the polyurethane and ensures a better crack closure.

## 1. Introduction

Concrete is vulnerable to crack formation due to its low tensile strength. Cracks in concrete may appear due to several causes such as drying shrinkage, external loading, temperature gradients, freeze–thaw action, *etc*. The presence of cracks leads to an accelerated ingress of water and other aggressive substances deep into the concrete matrix. In this way, harmful substances transported by the water reach the steel reinforcement very fast, which can lead to accelerated corrosion initiation and hence major durability problems.

To avoid problems regarding concrete deterioration and reinforcement corrosion, cracks need to be repaired as soon as possible. However, repair works require high direct and indirect costs and some cracked regions in concrete elements are not even visible or accessible. Therefore, it would be beneficial to give the concrete the ability to heal the cracks by itself without any human intervention. Different methods have already been explored to obtain autonomous crack healing in concrete [1]. One of the promising approaches is the embedment of brittle capsules filled with healing agents inside the concrete matrix [2,3,4]. Crack formation leads to capsule breakage and release of the healing agent, which fills up the crack and forms a barrier which prevents water ingress through the cracks.

Evaluation of the efficiency of the autonomous healing mechanism to prevent ingress of water through cracks can be done by water permeability tests [2,5,6] or gravimetrical water absorption tests [7,8,9]. Both test methods were originally designed to study uncracked, cracked and repaired cementitious materials [6,7,10,11,12,13,14,15,16,17,18]. From these tests the water permeability coefficient or sorption coefficient of specimens with healed cracks can be compared to specimens with unhealed cracks as well. This provides useful information regarding the healing efficiency. However, detailed information about the water ingress through healed cracks is not obtained in this way. In order to identify the difference in the development of the water distribution between sound, cracked and healed mortar or concrete, it is necessary to visualize the water flow. In this way, it becomes possible to evaluate whether the autonomous crack healing mechanism provides full sealing of the crack or whether there is still a part of the crack where water can enter. By visualizing the water uptake phenomenon over time, it also becomes possible to investigate whether the crack healing keeps preventing water ingress through the crack for long exposure periods to water. Interesting contributions regarding the visualization of the initial water uptake (up to 7 s) were published by Gardner *et al.* who studied the capillary rise heights for discrete cracks present in cementitious materials using a high speed digital camera [19]. Nevertheless, this technique cannot provide information regarding the water uptake in the matrix around the crack for longer exposure periods.

Radiation attenuation techniques such as X-ray and neutron radiography can be used for visualizing this water uptake in porous materials [20,21,22,23,24,25,26]. Both techniques are efficient to visualize water uptake processes, but X-rays interact differently with matter than neutrons. Heavy materials like steel or lead induce strong X-ray attenuation, whereas light materials like water or plastics result in a strong neutron attenuation. Since neutrons interact stronger with hydrogen than X-rays, neutron radiography is normally the best technique to visualize the moisture distribution in mortar samples.

Capillary water absorption in sound and cracked mortar and concrete has already been investigated in several studies by X-ray [9,27,28,29] or neutron radiography [17,30,31,32,33,34,35,36] experiments. In the research of Van Tittelboom *et al.* [30], the self-healing efficiency of mortar with encapsulated healing agents was visualized for the first time by neutron radiography. From the moisture distribution profiles on the neutron radiographs it was found that encapsulation of polyurethane-based healing agents proved to be very efficient for autonomous healing of single cracks.

The main aim of the current study was to investigate the efficiency of two polyurethane-based healing agents with different viscosity to prevent water ingress through cracks in mortar. Not only the effect of a single healed crack was studied in this research, also specimens with two cracks were produced to investigate the possibility of multiple crack healing. Moreover, in previous laboratory experiments, some time passed between triggering the autonomous crack healing mechanism and testing of the crack healing efficiency to ensure hardening of the healing agent. However, in real structures cracks can appear during periods where the structure is already exposed to water. Therefore, the effect of instant autonomous crack healing during water exposure was also studied.

## 2. Materials

### 2.1. Mortar

Mortar with a water-to-cement ratio of 0.5 and a sand-to-cement ratio of 3 was made. Sand with grain sizes ranging from 0 to 2 mm was used in combination with Ordinary Portland cement CEM I 52.5 N. The proportioning of the different constituents per m³ of mortar was as follows: 1535 kg/m^3^ sand, 512 kg/m^3^ cement and 256 kg/m^3^ water.

### 2.2. Capsules

In order to encapsulate the selected healing agents, borosilicate glass capsules with a length of 30 and 50 mm, an internal diameter of 3.00 mm and an external diameter of 3.35 mm were chosen. As glass is a very brittle material, breakage of the capsules upon crack creation will be assured.

### 2.3. Healing Agent

Two different types of polyurethane-based healing agents were selected for this study. As the viscosity of the healing agent is a very important parameter that determines whether the healing agent will flow out of the capsules and fill the crack, healing agents with different viscosities were chosen. The first agent was developed within the framework of the SHEcon project (Self-healing concrete for structural and architectural applications) where a polyurethane-based healing agent was developed in order to meet the needs for self-healing of thermally induced cracks in concrete sandwich panels [37,38]. It has a viscosity of 6700 mPas at 25 °C. It is a polyurethane (PU) precursor that essentially consists of methylene diphenyl diisocyanate (MDI) and a polyether polyol. This precursor reacts with water to create the foam that heals the cracks. The moisture content of the concrete itself counts as the main water source. The second healing agent is a commercially available healing agent named Flex SLV AF from the company De Neef Conchem with a much lower viscosity of about 200 mPas at 25 °C. It is a MDI and polyether polyol based prepolymer. As such, the backbone of the product is an incomplete PU polymer containing residual isocyanate (-NCO) groups that react with the water present in the mortar upon capsule breakage to form a waterproof PU polymer. The healing agent is free of volatile organic compounds, does not contain catalysts or any water-soluble products. The viscosity and rheological behavior upon release from the capsules is mainly controlled by the presence of inert, hydrophobic compounds. The polyurethane-based healing agent with the highest viscosity is named PU_HV and the one with the lowest viscosity PU_LV. Both healing agents are one-component healing agents that react upon contact with moisture in the matrix. Upon their reaction, foaming occurs which causes expansion of the healing agent. This is desirable to fill the crack space completely and assure that the crack is sealed against the ingress of aggressive substances.

### 2.4. Mortar Prisms with(out) Encapsulated Healing Agent

Table 1 gives an overview of all studied test series of mortar prisms together with their nomenclature and the number of specimens per test series. Prisms with dimensions of 40 × 40 × 160 mm^3^ were prepared by using wooden molds. In order to create standardized cracks (see Section 3.1), thin metal plates were positioned in the molds up to a depth of 20 mm. These plates had a width of 40 mm, a thickness of 300 µm and were fixed at their position by means of a metal framework that was connected to the mold. In order to evaluate the healing efficiency of the proposed self-healing mechanism, one test series without inserted metal plates, and thus without standard cracks, was provided (UN). The samples of all other test series did contain one or two metal plates and thus one or two standard cracks. For samples with only one crack (CR_1), the metal plate was positioned at the center of the samples’ length. For samples with two cracks (CR_2), both plates were positioned in parallel with an intermediate distance of 20 mm symmetrically, with regard to the center of the sample.

**Figure 1:**
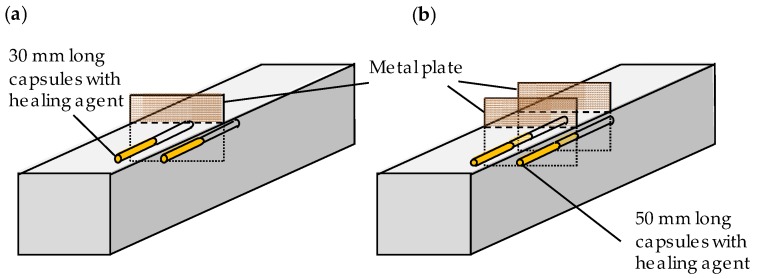
Mortar samples with autonomous crack healing properties containing: one single crack (**a**); and two cracks (**b**).

Samples with self-healing properties were obtained by embedding encapsulated healing agent in the matrix at the position where the crack would appear. In order to trigger the healing mechanism at the moment of crack appearance, a method described by Van Belleghem *et al.* [9] was used. Since standardized cracks were created, the capsules were put through the metal plates used for crack creation. Therefore, two holes with a diameter of 3.50 mm were drilled in the metal plates that were used to create the cracks in the samples with self-healing properties. The capsule’s position (and thus also the position of the holes) was chosen in such a way that there was a mortar cover on each capsule of 10 mm at the side and the bottom (top in Figure 1) of the sample. The 30 and 50 mm length capsules were introduced in the samples containing one and two plates, respectively, and positioned through the holes inside the metal plate(s). Once the capsules were placed through the holes in the plates, their position was fixed by gluing them onto thin plastic wires, which were connected to the walls of the mold (Figure 1).

Once preparation of the molds was finished, samples were made by filling the molds with mortar in two equal layers. Each layer was compacted through vibration on a vibrating table. After casting, the samples were stored in an air-conditioned room at a temperature of 20 °C and a relative humidity of more than 95%. Twenty-four hours later, samples were demolded and then stored again in the same air-conditioned room until the age of 28 days.

## 3. Methods

### 3.1. Crack Creation and Healing

For the samples containing cracks without healing agent (CR_1 and CR_2), the metal plates were removed from the mortar matrix at the moment of demolding. In this way, standard cracks with a depth of 20 mm, a length of 40 mm and a width of 300 µm were created. For all samples with self-healing properties, the metal plates were removed after the curing period of 28 days, except for two samples of both the CR_1_PU_HV and CR_1_PU_LV series (specimens D and E). For these last four samples, cracks were created and thus healing was triggered just before exposure to the water absorption test. This was done in order to investigate the healing efficiency of the samples if cracking occurs upon exposure to water and thus the healing agent had not yet hardened in the crack at the moment of contact with water.

Autonomous crack healing was obtained for the samples with embedded capsules as the capsules were broken at the position of the crack plane at the moment that the metal plates were removed. Breakage of the capsules was followed by release of the healing agent out of the capsules, which developed into the cracks. Due to contact of the healing agent with moisture inside the cementitious matrix, the healing agent started to polymerize, resulting in crack repair.

### 3.2. Sample Preparation

As the crack creation technique with metal plates required the test surface to be the troweled surface, the test surface of the samples was rough. In order to obtain a flat test surface, a layer of approximately 1–2 mm was cut off.

Neutron radiography images are produced by radiation passing through an object. The spatial resolution of the radiographs therefore depends on the thickness of the scanned object. In the case of mortar, a sample thickness of 40 mm is quite high in order to obtain a good spatial resolution of the water content in the samples. Therefore, all specimens were sawn along the longitudinal axis in two equal parts. The two halves had a thickness ranging from 17 to 20 mm. The exact thickness of each half was measured with a caliper with an accuracy of 0.05 mm. One half of each sample was used for the water absorption tests.

**Figure 2:**
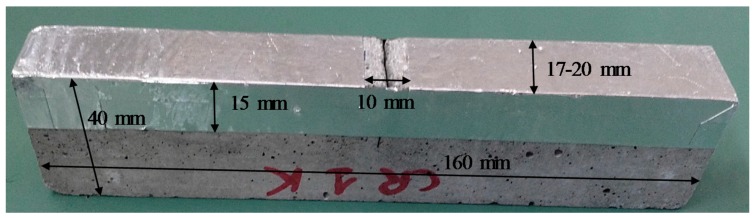
Mortar sample, with one standard crack (CR_1), covered with aluminum tape.

All specimens were dried in an oven at 40 °C until constant mass (mass change less than 0.1% in 24 h) was achieved. In this way, a uniform moisture distribution was obtained inside the sample in a short time period. After the drying period, the sides of the specimens were covered over a height of 15 mm with a self-adhesive aluminum tape so that the water could only enter the samples through the test surface (top surface in Figure 2). Regular aluminum tape was used since it interacts only weakly with neutrons (it is almost transparent for neutrons). Since the main aim of the research was the investigation of the water uptake in the crack region, a large part of the bottom surface was also covered with the aluminum tape so that only a 10 mm wide zone around the cracks was exposed to water during the absorption test (Figure 2).

### 3.3. Visualization of Water Ingress

#### 3.3.1. Neutron Radiography Facility

The visualization of the water ingress in cracked mortar was performed by real time neutron radiography at the thermal neutron imaging facility NEUTRA. This facility is part of the spallation neutron source SINQ of the Paul Scherrer Institute (PSI) in Switzerland. The neutron beam of the spallation source was guided to a fixed size aperture of 2 cm in diameter by means of a convergent inner collimator tube. From there, a divergent outer collimator led the parallelized neutron beam to the object space with a useful area of 400 mm diameter. The thermal energy spectrum of the neutron beam was characterized by a Maxwell–Boltzmann distribution with peak energy of 25 meV. The neutron beam then passed through the studied samples to a 100 µm thick LiF/ZnS scintillator screen. This scintillator converted the neutrons to visible light, which was deflected by mirrors in a dark room and recorded by a cooled slow-scan Andor SCMOS camera with a 50 mm AF-S NIKKOR lens.

For each series of specimens, open beam, dark current and black body images were acquired. The open beam image represents the spatial distribution of the neutron beam intensity and aimed at removing the inhomogeneity of the beam. The dark current image was taken while all shutters of the beam line were closed. It was used as a correction for the background noise level of the camera. The black body image was obtained by placing neutron absorbing blocks of boronated polyethylene in front of the scanned samples. This was used as a background scattering correction.

#### 3.3.2. Water Uptake during Scanning

**Figure 3:**
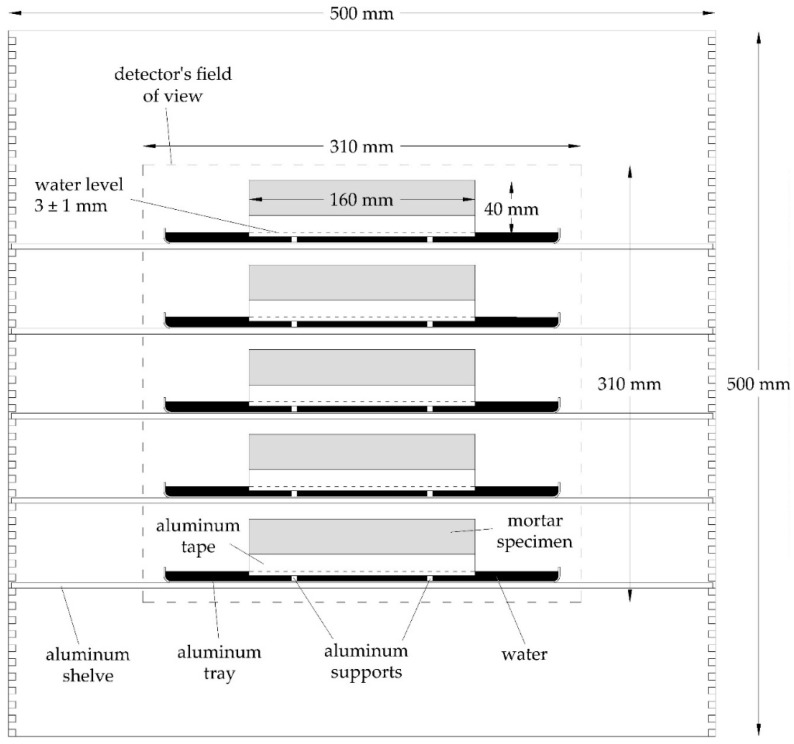
Setup of the samples in the aluminum test frame during scanning.

Five samples could be scanned simultaneously since the detector’s field of view measured 310 × 310 mm^2^. Therefore, a test frame with five aluminum shelves was used (Figure 3). Aluminum containers were placed on each of the shelves. The mortar specimens were placed on aluminum line supports in the containers. A reference image of the samples in the dry state was taken before the containers were filled with water. Water was manually added to the containers by means of a syringe so that the immersion of the samples amounted to 3 ± 1 mm. When all five containers were filled, radiographs were taken with an exposure time of 3 s, which resulted in a time step of 4.6 s due to a process delay. This resulted in a pixel size of 0.273 mm for all images. Radiographic scanning during the water absorption process continued for 2–4 h.

### 3.4. Image Analysis

In order to be able to accurately analyze the moisture distribution profiles in the samples, the corrections mentioned in Section 3.3.1 needed to be taken into account. Dark current image and black body image were subtracted from each of the neutron radiographs in order to take into account the background noise of the camera and the background scattering. The same was done for the open beam image. Next, a flat field correction was applied by dividing the corrected neutron radiographs by the corrected open beam image. All image operations were performed using the image processing software ImageJ (1.48v, National Institutes of Health, Bethesda, MD, USA).

Visualization of the water front was done by dividing the images in the wet state by the reference image in the dry state. The moisture profile in the mortar samples was visualized in this way for three different exposure times: 5 min, 30 min and 4 h. From these images the crack healing with different polyurethanes could be evaluated at different time steps. Since the inclusion of the images for all samples would make this paper too lengthy, only the imaging of one representative specimen per test series was included.

**Figure 4:**
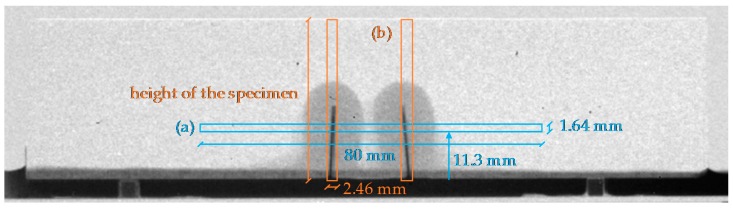
Location and dimensions of the rectangular areas for water profiling in a sample neutron radiography image: (**a**) perpendicular to the cracks; and (**b**) coinciding with the cracks.

Next to the visual evaluation, a quantitative evaluation of crack healing was performed by plotting the water profiles of the obtained images at three different times: 5 min, 30 min and 4 h. Calculation of the water content was done in a similar way as described previously [30]. Horizontal water profiles were determined in the central 80 mm of each specimen at a height of 11.3 mm above the bottom of the samples. Figure 4 shows the as such defined rectangular area for water profiling in a schematic way for a sample neutron image. The height of the rectangular area perpendicular to the cracks (Figure 4a) was chosen so that the profile was always determined above the capsule layer of the self-healing specimens. In this way, the presence of the capsules did not interfere with the water profile in the mortar. Water profiles were also taken along the cracks (Figure 4b). Therefore, additional rectangular areas were defined that coincided with the cracks and extended from the bottom to the top of the sample.

### 3.5. Analysis of the Spread Region of the Healing Agents in the Crack

After visualization of the water uptake by neutron radiography, the specimens with healed cracks were split in order to analyze the spread region of the polyurethanes on the crack faces. In this way, the amount of water uptake through the healed cracks could be linked to the amount of polyurethane that filled up the crack. For the healed specimens containing two cracks, the specimens were split twice so that both cracks could be analyzed. Hence, it could be determined whether the healing agents were equally distributed in both cracks.

## 4. Results and Discussion

### 4.1. Neutron Radiography Imaging of Crack Healing

#### 4.1.1. (Un)cracked Condition

**Figure 5:**
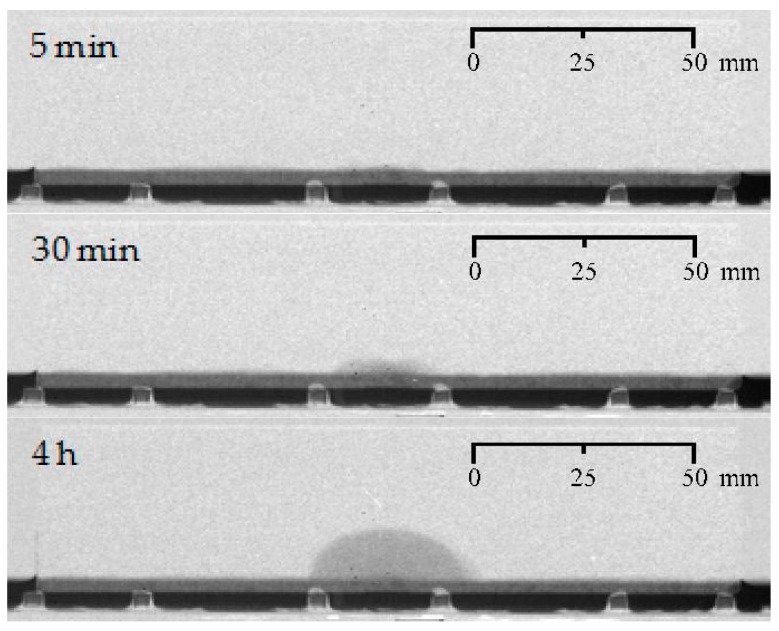
Visualization of the water uptake as a function of exposure time using neutron radiography for one uncracked mortar prism (representative sample: UN_A).

Before going into detail on the ability of an encapsulated PU-based healing agent to prevent accelerated water ingress in cracks, the water uptake by capillary action of uncracked and cracked mortar was studied. When looking at the water uptake of a representative uncracked specimen as a function of exposure time (Figure 5), it is clear that the water front only very gradually progressed starting from the 10 × 20 mm^2^ exposed area at the bottom surface of the prism. It took 30 min before the height of the water front actually rose above the water level in the tray. After having been in contact with water for 4 h, the maximum height of the water front amounted to 14 mm starting from the bottom surface of the specimen or 11 mm above the water level in the tray. The maximum width of the lateral water front was around 35 mm. The recorded water ingress through the undamaged cementitious matrix is expected to be present also in the artificially cracked samples, but it will of course be surmounted with additional ingress, which is entirely induced by the presence of the crack.

**Figure 6:**
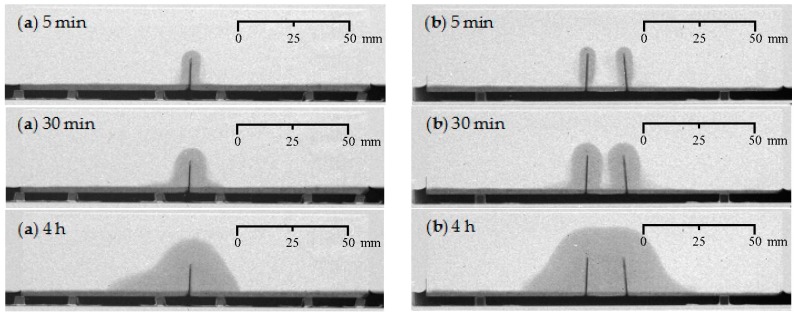
Visualization of the water uptake as a function of exposure time using neutron radiography for cracked mortar prisms: (**a**) one artificial crack (representative sample: CR_1_B); and (**b**) two artificial cracks (representative sample: CR_2_C).

With one or two articial cracks present (Figure 6), logically, there is a faster rise of the water front above the water level in the tray from the start of the experiment onwards due to capillary suction phenomena at the location of the standardized crack(s).

After 4 h of exposure, the maximum height of the water front in the mortar prism containing just one crack (Figure 6a) was 27 mm. When the crack tip is taken as reference point, the water front is extending around 12 mm in upward direction. In the lateral direction, the water uptake in the vicinity of the crack did not turn out to be completely symmetrical. The final lateral ingress on the left hand side seems to extend further, but also appears a bit more vague at the very end. Most probably, the aluminum tape which was applied on the bottom surface of the specimen next to the 10 × 20 mm^2^ exposure area, was not perfectly attached to the mortar surface. This allowed for some additional water ingress in between the tape and the specimen. By multiplying the more correct lateral water front at the right hand side of the crack by two, a more reliable indication of the maximum overall lateral width of the water front can be obtained. It should be equal to 47 mm. Thus, when comparing the dimensions of the final lateral water fronts of the uncracked and cracked samples, being 35 mm *versus* 47 mm, the undesirable effects of the crack are quite significant. Comparison of the maximum heights of the water fronts for uncracked and cracked mortar, being 14 mm *versus* 27 mm from the bottom of samples, leads to a similar conclusion.

For the specimen with two cracks (Figure 6b), the initial water uptake per crack is very similar to that of the specimen with a single crack. However, after 30 min of exposure, the water fronts around the two individual cracks are about to merge. From then onwards their combined effect should be taken into consideration. This time, the overall water front stayed quite symmetrical until the very end of the experiment. The final maximum rise of the water front starting from the crack tips amounted to around 13 mm, while the overall lateral water front was around 80 mm in width near the bottom of the specimen. The water front at the left hand side of the left crack and the right hand side of the right crack extended for about 30 mm outwards on both sides. This is more than for the specimen containing just one crack (Figure 6a). There, around 23 mm was measured for the more accurate maximum ingress at the right hand side of the crack.

#### 4.1.2. Healing of One Crack

**Figure 7:**
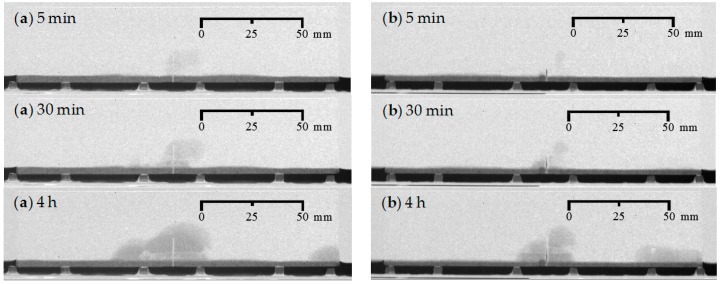
Visualization of the water uptake as a function of exposure time using neutron radiography for mortar prisms with one crack healed: (**a**) crack healing with PU_HV (representative sample: CR_1_PU_HV_A); and (**b**) crack healing with PU_LV (representative sample: CR_1_PU_LV_A).

Comparison of the water fronts observed for the samples where one crack was healed with an encapsulated PU-based healing agent (Figure 7) with those of the (un)cracked mortar (Figure 5 and Figure 6a), allows a thorough evaluation of the crack healing ability.

The water uptake of the specimens just after bringing them in contact with the water (after 5 min) is clearly much less than when an unhealed crack was present. In other words, the release of the PU upon capsule breakage was able to eliminate the strong capillary suction effects in the artificial cracks quite properly. This statement holds true for the two PU-based healing agents, either with a high or a low viscosity (PU_HV and PU_LV). Also note that the white grey color inside the crack represents the polyurethane and thus indicates a substantial filling of the crack.

With increasing exposure time, a water front above the water level gradually becomes visible in the vicinity of the crack. In terms of grey value it is much vaguer than for the cracked samples. Still, this water front progresses faster than in case of the uncracked sample. After 30 min, it is clearly more visible, while for the uncracked sample it takes longer before something can be seen above the water level. By the end of the experiment, there is an overall water front that is clearly larger in area than in case of the uncracked sample. This is especially the case for the sample containing the high viscosity PU-based healing agent (Figure 7a). Based on a mainly visual evaluation on one sample, the low viscosity PU-based healing agent seemed to have worked more effectively (Figure 7b, PU_LV).

Note that for the sample containing encapsulated PU_LV (Figure 7b) there are traces of undesired water sorption at the right hand side of the sample. This is merely due to an improper attachment of the aluminum tape onto the bottom surface of the mortar prism. Although the aluminum tape was always carefully applied, this problem could not always be avoided unfortunately.

#### 4.1.3. Healing of Two Cracks

**Figure 8:**
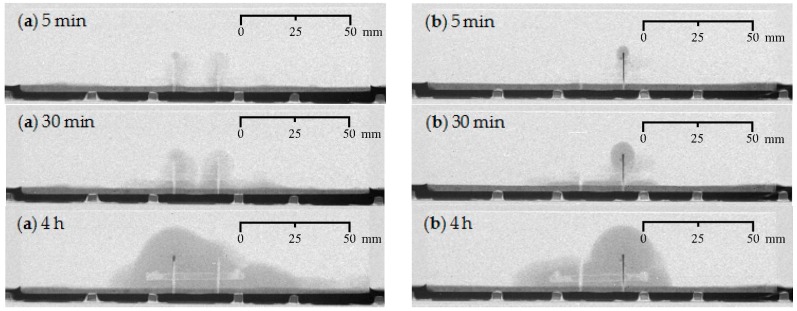
Visualization of the water uptake as a function of exposure time using neutron radiography for mortar prisms with two cracks healed: (**a**) crack healing with PU_HV (representative sample: CR_2_PU_HV_C); and (**b**) crack healing with PU_LV (representative sample: CR_2_PU_LV_B).

For the specimens with self-healing properties containing two artificial cracks, the following conclusions can be drawn (Figure 8). When using a high viscosity PU for healing purposes (Figure 8a, PU_HV), the initial water uptake after 5 min in the vicinity of the cracks is substantially reduced for both cracks. As time passes, the progressing water front shows that the crack on the right hand side is healed better than the one on the left hand side. Thus, the healing performance for the two cracks is not equal. The same goes for the specimen that contained a 50 mm long capsule filled with a low viscosity PU precursor (Figure 8b, PU_LV). The only difference is that the unequal water uptake for the two cracks is already visible just after starting the experiment. The crack on the right hand side barely shows signs of healing, while the PU in the one on the left hand side seems to be preventing water ingress almost completely, even after having been in contact with water for 4 h.

The difference in behavior between the two PU-based healing agents with respect to the proper healing of two cracks could maybe be attributed to their difference in viscosity. When subsequently pulling out the two thin metal plates (within a timespan of less than one minute) to break the capsules and initiate the release of the healing agent, the high viscosity PU precursor tends to flow quite easily into the two cracks. On the other hand, the low viscosity PU precursor rather flows into the crack that corresponds with the thin metal plate that was pulled out first. Due to the lower viscosity, hence greater fluidity, PU_LV seeped more quickly into one of the cracks before extracting the second metal plate and thereby leaving less material to fill the second crack. Whereas in the specimen containing the high viscosity healing agent, the extraction of the metal plates was relatively fast to allow a more even distribution of the agents along the two cracks. However, careful evaluation of the other specimens that were tested cannot unambiguously confirm this assumption. For a more in-depth evaluation on that matter, we refer to Section 4.4 where the spread region of the self-healing substances onto the crack walls has been assessed experimentally. Another general observation that can be made is that the amount of PU-based healing agent present in 50 mm long glass capsules is insufficient to properly heal two adjacent cracks. This is quite logic as the PU amount present in one 30 mm long capsule was not enough to fully prevent the water ingress in just one crack.

#### 4.1.4. Water Front/Sample Cross-Section Area Ratios

The water front/sample cross-section area (WFA/SCA) ratios as calculated in ImageJ (1.48v, National Institutes of Health, Bethesda, MD, USA) from the neutron radiography images after 4 h of exposure (Figure 5, Figure 6, Figure 7 and Figure 8), allow a somewhat more quantitative evaluation of the healing ability when using the two considered types of encapsulated PU-based healing agent (Table 2).

When comparing the uncracked test series (UN) and the test series containing just one crack (CR_1), the mean WFA/SCA ratio of the latter is higher. Nevertheless, the rather high standard error (5.0%) for the uncracked series indicates that the water uptake could be as high as for cracked mortar. This rather unexpected observation can mainly be attributed to the fact that the aluminum tape at the bottom surface of one of the uncracked samples (UN_B) was not entirely well attached and therefore caused some undesirable extra water uptake (WFA/SCA: 15.8%) through capillary suction phenomena between the tape and the bottom surface. Sample UN_A was characterized by a WFA/SCA ratio of only 5.8%. In comparison with that value, the effect of one crack on the water uptake is evident (WFA/SCA: 14.1%). The self-healing test series with one crack (CR_1_PU_HV and CR_1_PU_LV) have WFA/SCA ratios that are slightly higher than 14.1%, *i.e.*, 16.7% and 15.7%, respectively. This would indicate that the healing mechanism was not effective at all. Nonetheless, their high standard errors (3.5% and 4.5%, respectively) demonstrate that considerably lower values are very well possible. The recorded minimum values amounted to 11.9% and 6.9%, respectively. Comparison of those two values would indicate a slight preference for the low viscosity healing agent. However, given their high susceptibility to variation, it remains difficult to draw definitive conclusions on that matter.

Evaluation of this parameter for the specimens in which two cracks should have healed (CR_2_PU_HV and CR_2_PU_LV), shows that for both the high and low viscosity PU-based healing agents the WFA/SCA ratios (25.3% and 24.0%) are only slightly lower compared to the cracked condition (CR_2: 26.9%). Given the substantial standard error on those results (3.1% and 3.8%, respectively) the differences with the cracked reference (CR_2) are almost non-existing. The amount of PU precursor incorporated in the 50 mm long capsules flowing into the cracks is not sufficient to establish a decent healing performance. Based on the WFA/SCA ratios, a higher dosage of healing agent would be preferred. Again, the WFA/SCA ratios do not indicate a preference for a high or low viscosity healing agent. On the other hand, one should be aware of the fact that a low viscosity healing agent mainly flows towards one crack, the one that has been created first. This was clearly visible in the neutron radiography images (Figure 8b). Thus, for multiple crack healing, a low viscosity PU-based healing agent may not necessarily be an advantage, depending on the expected crack distance. A higher dosage of PU_HV may be recommended in that case.

### 4.2. Water Profiling of Crack Healing Perpendicular to the Crack(s)

#### 4.2.1. (Un)cracked Condition

**Figure 9:**
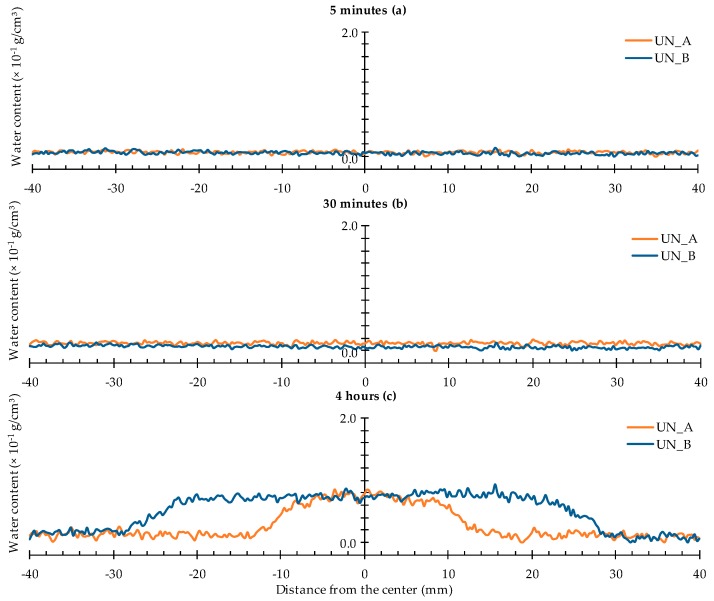
Water profiles of the two uncracked standard mortar prisms: (**a**) after 5 min; (**b**) after 30 min; and (**c**) after 4 h.

The water profiles corresponding with a rectangular area taken at a height of 11.3 mm above the bottom of the samples (Figure 9), lead to quite similar conclusions as the neutron radiography images shown in Section 4.1.1.

After 30 min of exposure, there is still no increased water content at the selected zone in the sample, whereas, by the end of the experiment, it showed an increase of 0.8 × 10^−1^ g/cm^3^. The increased water content is mainly located around the central 10 × 20 mm^2^ exposed area. The distance from the expected location of a crack ranges from (−10, 10 mm) to (−30, 30 mm).

Note that the water profiles of samples UN_A and UN_B after 4 h of exposure differ significantly. The broad water spread in case of the latter sample is most probably due to the fact that the aluminum tape did not attach entirely well to the bottom surface of that mortar prism. The water profile of sample UN_B should therefore be interpreted with caution.

**Figure 10:**
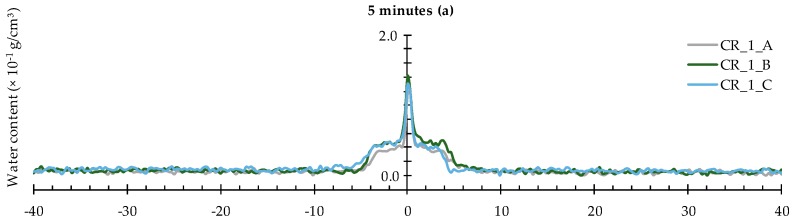

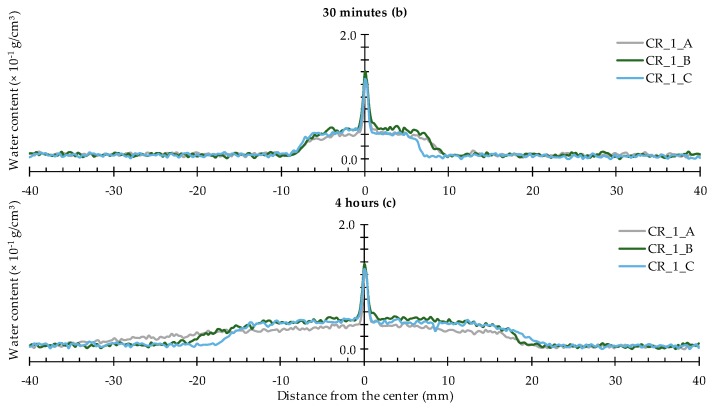
Water profiles of the three standard mortar prisms containing one artificial crack: (**a**) after 5 min; (**b**) after 30 min; and (**c**) after 4 h.

In the presence of one artificial crack, the profiles show an obvious steep peak corresponding with the water absorbed by capillary action in the crack itself (Figure 10). The notable water profile adjacent to this peak represents the water uptake by the mortar matrix near the crack wall. This water profile clearly progresses laterally as a function of exposure time (Figure 10a–c) while the peak corresponding with the crack did not show significant change.

**Figure 11:**
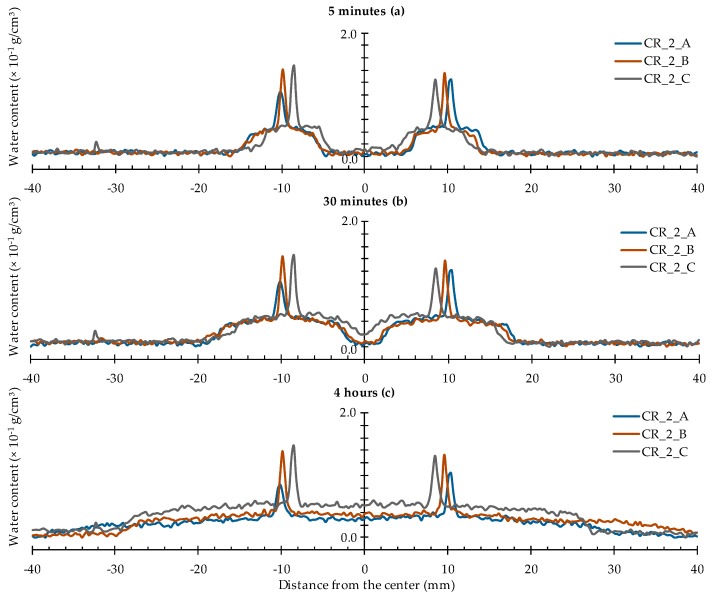
Water profiles of the three standard mortar prisms containing two artificial cracks: (**a**) after 5 min; (**b**) after 30 min; and (**c**) after 4 h.

For the specimens containing two cracks (Figure 11), the obtained water profiles can be more or less considered as a superposition of two profiles of a singular crack. Only after about 30 min of exposure the effect of the merging of the two profiles should be taken into consideration as well. Just like for the specimens with one crack, the water profiles mainly extend in the lateral direction as function of exposure time.

#### 4.2.2. Healing of One Crack

**Figure 12:**
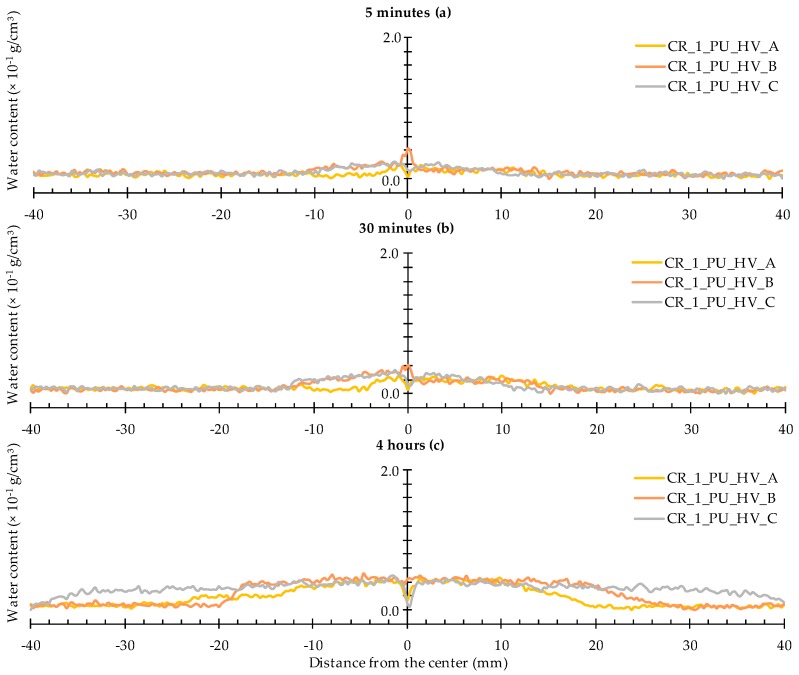
Water profiles of the three standard mortar prisms containing one artificial crack healed with PU_HV: (**a**) after 5 min; (**b**) after 30 min; and (**c**) after 4 h.

**Figure 13:**
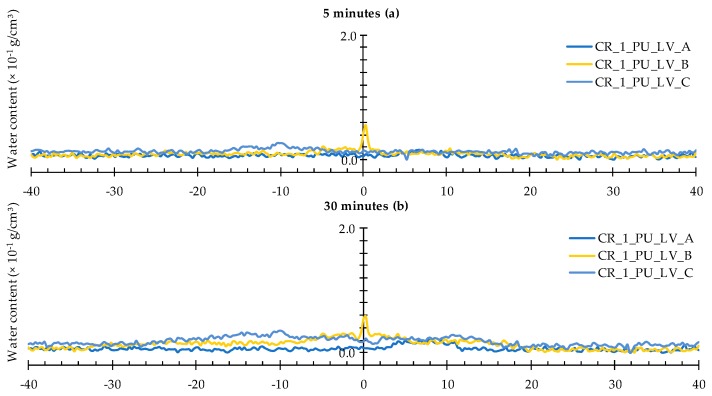

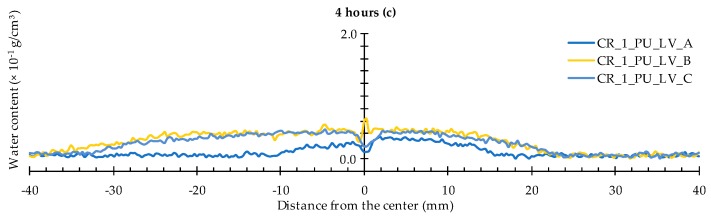
Water profiles of the three standard mortar prisms containing one artificial crack healed with PU_LV: (**a**) after 5 min; (**b**) after 30 min; and (**c**) after 4 h.

When looking at the water profiles for the rectangular area above the position of the capsule, little difference was observed between the specimens that were healed with the high and low viscosity healing agent (Figure 12 and Figure 13).

Opposite results were observed when comparing to those of the neutron radiography images of samples CR_1_PU_HV_A and CR_1_PU_LV_A. There, the water fronts exhibited by the former sample looked more pronounced than in the latter. However, this is not completely unexpected as the calculated water profiles only relate to a very thin rectangular area above the capsule while the visually observed water front in the neutron radiography images relates to the whole cross-sectional area around the crack zone. Still, one should be aware of the fact that the neutron images in Figure 7 do not account for the possible variation within each of the two specimen groups (CR_1_PU_HV and CR_1_PU_LV). Additional consideration of the neutron radiography image related mean WFA/SCA ratios and their corresponding standard errors (Table 2) indicates no preference for the high or low viscosity healing agent. This is completely in line with the water profile results.

A closer look at the individual profiles shows that the crack itself was not always properly healed. For each type of healing agent (PU_HV and PU_LV), one out of three samples still shows an upward peak indicating presence of water in the crack. Ideally, a healed sample should be characterized by a significant downward peak. The more the downward peak reaches the flat baseline curve for an unexposed area of the sample, the lower the amount of water. Given this, there is an obvious healing effect for two out of three samples per type of healing agent. Still, it must be said that the expected enhanced healing performance in comparison to the observed was actually higher.

#### 4.2.3. Healing of Two Cracks

**Figure 14:**
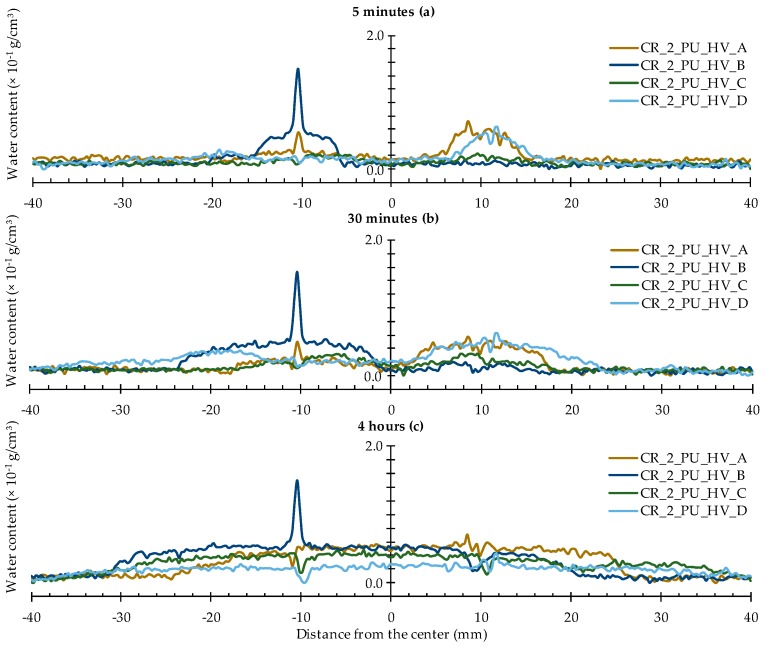
Water profiles of the four standard mortar prisms containing two artificial cracks healed with PU_HV: (**a**) after 5 min; (**b**) after 30 min; and (**c**) after 4 h.

**Figure 15:**
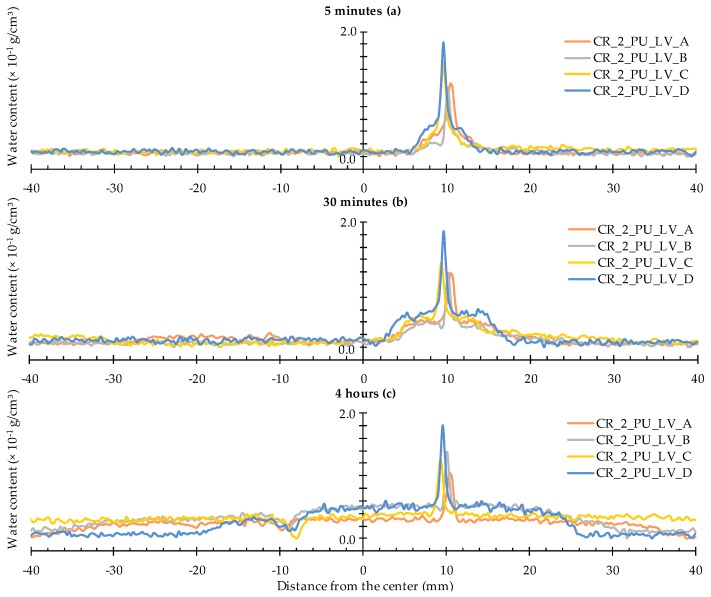
Water profiles of the four standard mortar prisms containing two artificial cracks healed with PU_LV: (**a**) after 5 min; (**b**) after 30 min; and (**c**) after 4 h.

The water profiles for the specimens with two healed cracks (Figure 14 and Figure 15) are to be interpreted in a similar way by looking for the upward and downward peaks and how they evolve. From the comparison of the neutron radiography images of samples CR_2_PU_HV_C and CR_2_PU_LV_B (Figure 8), it seemed that with PU_HV, the two cracks were somewhat more equally filled than with PU_LV, especially in the initial stages of the experiment. The water profiles obtained for each test series of samples further confirm this. Except for the water profile of specimen CR_2_PU_HV_B, major upward peaks are completely absent in the water profiles of the other replicates containing PU_HV. On the other hand, the downward peaks do not always completely extend to the baseline of the water profiles.

With PU_LV, a substantial upward water content related peak can be seen in one crack for all four tested samples. The peak water content in all samples is related to the crack which appeared second in the sample. Thus, the PU_LV tends to flow mainly into the crack that was created first, leaving the other one practically unhealed. Evaluation of the water profiles along the cracks and the spread region of the self-healing substances on the crack faces further confirms this (see Section 4.3 and Section 4.4). It is also worth noticing that the downward peaks after 4 h of exposure in the one healed crack seem a bit more pronounced than when using PU_HV.

#### 4.2.4. Water Profile Integrals

The integrals of all previously shown water profiles (Figure 9, Figure 10, Figure 11, Figure 12, Figure 13, Figure 14 and Figure 15, area in between the peaks and the baseline of the profiles), give an indication of the healing efficiency of the different samples for a very localized rectangular area at a height of 11.3 mm from the samples’ bottom surface. The mean values of these integrals and the corresponding standard errors have been summarized in Table 3.

On the local scale, *i.e.*, the thin 80 mm long rectangular area above the capsule near the crack tip, there is a marginal healing effect of incorporating capsules filled with PU_HV or PU_LV to heal one or two cracks. The integrals calculated for the samples with self-healing properties do not differ significantly from the cracked ones. Moreover, based on these results little difference was observed in terms of healing ability depending on whether the polyurethane precursor with the high or low viscosity was used. This is in line with the previously recorded findings of the mean WFA/SCA ratios and their corresponding standard errors (Table 2). Both approaches indicate that the matrix around the crack(s) can still be quite affected by the presence of the crack in terms of water ingress even after (partial) healing. Within the cracks, the healing ability can often be considered more adequate. The water profiles along the cracks that are shown in the next section (Section 4.3) prove this. Nonetheless, the healing performance with encapsulated PU precursors could still be improved substantially, at least in the case where the specimens were exposed to water only after the healing agents had been able to react to a maximum extent with the moisture content of the surrounding mortar and the air humidity.

### 4.3. Water Profiling of Crack Healing along the Crack(s)

#### 4.3.1. Cracked Condition

**Figure 16:**
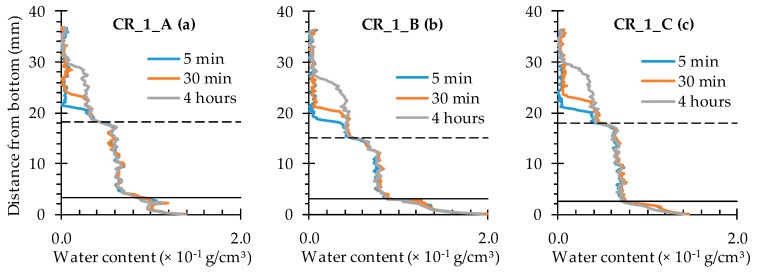
Water profiles along the crack as a function of exposure time for the neutron radiography images of the three standard mortar prisms containing one artificial crack: (**a**) CR_1_A; (**b**) CR_1_B; and (**c**) CR_1_C.

**Figure 17:**
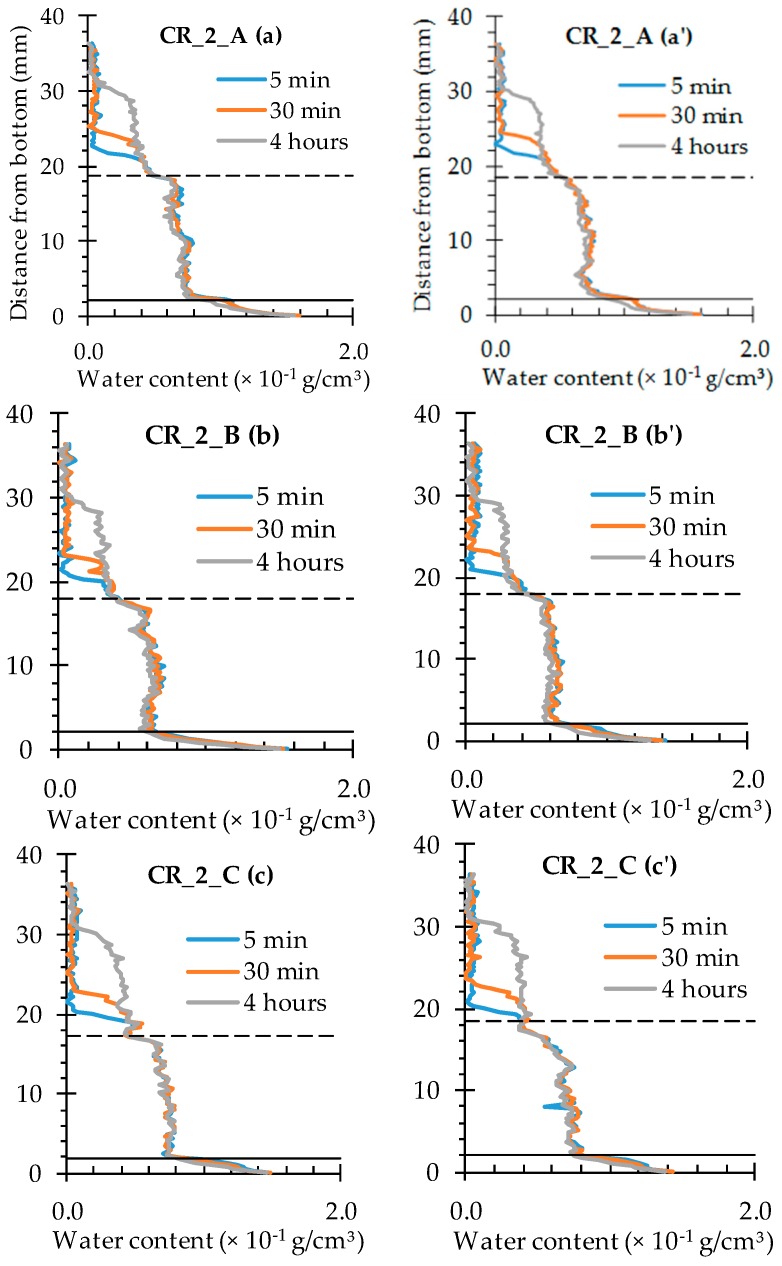
Water profiles along the crack as a function of exposure time for the neutron radiography images of the three standard mortar prisms containing two artificial cracks: (**a**) CR_2_A crack on the left; (**a’**) CR_2_A crack on the right; (**b**) CR_2_B crack on the left; (**b’**) CR_2_B crack on the right; (**c**) CR_2_C crack on the left; and (**c’**) CR_2_C crack on the right.

All water profiles taken along the cracks of the cracked samples without self-healing properties look very similar (Figure 16 and Figure 17). At the crack location (the graph sections in between the full and the dashed horizontal black lines), the water content amounts to 0.6–0.8 × 10^−1^ g/cm^3^. This water content range is maintained over the entire crack depth and does not really change as a function of exposure time. On the other hand, above the crack tips (the graph sections above the dashed horizontal black lines) the water content obviously increases with time. After only 5 min of exposure, an increased water content in the matrix of around 0.4 × 10^−1^ g/cm^3^ can only be observed up to a sample height of 20 mm. After 30 min, the affected sample height is only slightly higher, while a 4-h exposure period resulted in a 0.4 × 10^−1^ g/cm^3^ water content up to a sample height of 30 mm. These observations are valid for the individual water profiles along the crack for both the specimens containing one and two artificially induced standardized cracks.

#### 4.3.2. Healing of One Crack

**Figure 18:**
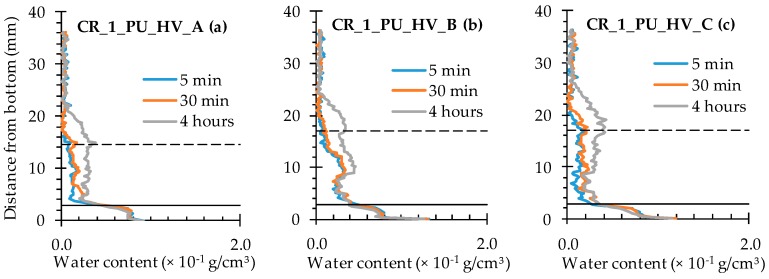
Water profiles along the crack as a function of exposure time for the neutron radiography images of the three standard mortar prisms containing one artificial crack healed with PU_HV: (**a**) CR_1_PU_HV_A; (**b**) CR_1_PU_HV_B; and (**c**) CR_1_PU_HV_C.

**Figure 19:**
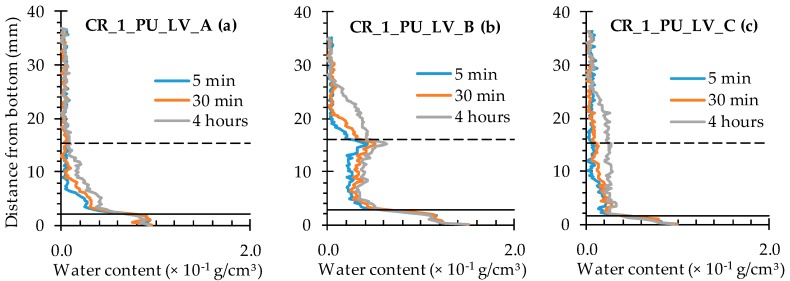
Water profiles along the crack as a function of exposure time for the neutron radiography images of the three standard mortar prisms containing one artificial crack healed with PU_LV: (**a**) CR_1_PU_LV_A; (**b**) CR_1_PU_LV_B; and (**c**) CR_1_PU_LV_C.

In comparison with the water profiles along the crack for the cracked specimens (Figure 16), the ones corresponding to the samples containing encapsulated PU_HV (Figure 18) and PU_LV (Figure 19) are characterized by a substantially reduced water content in between the full and the dashed horizontal black lines of the graphs. In other words, there is a significant healing effect for the crack itself. Nevertheless, this reduced water content of around 0.2 × 10^−1^ g/cm^3^ still tends to increase a bit with exposure time to around 0.4 × 10^−1^ g/cm^3^ after 4 h. The evolution in water content with exposure time and sample height also still exists for the sample matrix above the crack tip. Only now, the affected sample height is clearly lower than 30 mm (maximum 26 mm). The most promising water profiles in terms of healing efficiency were obtained for samples CR_1_PU_LV_A and CR_1_PU_LV_C (Figure 19). Such low water contents could not be achieved by using encapsulated PU_HV (Figure 18). This does not necessarily mean that the low viscosity healing agent should have the preference because the third sample of the series (CR_1_PU_LV_B) clearly has a higher water content and even shows signs of water enrichment near the crack tip.

#### 4.3.3. Healing of Two Cracks

**Figure 20:**
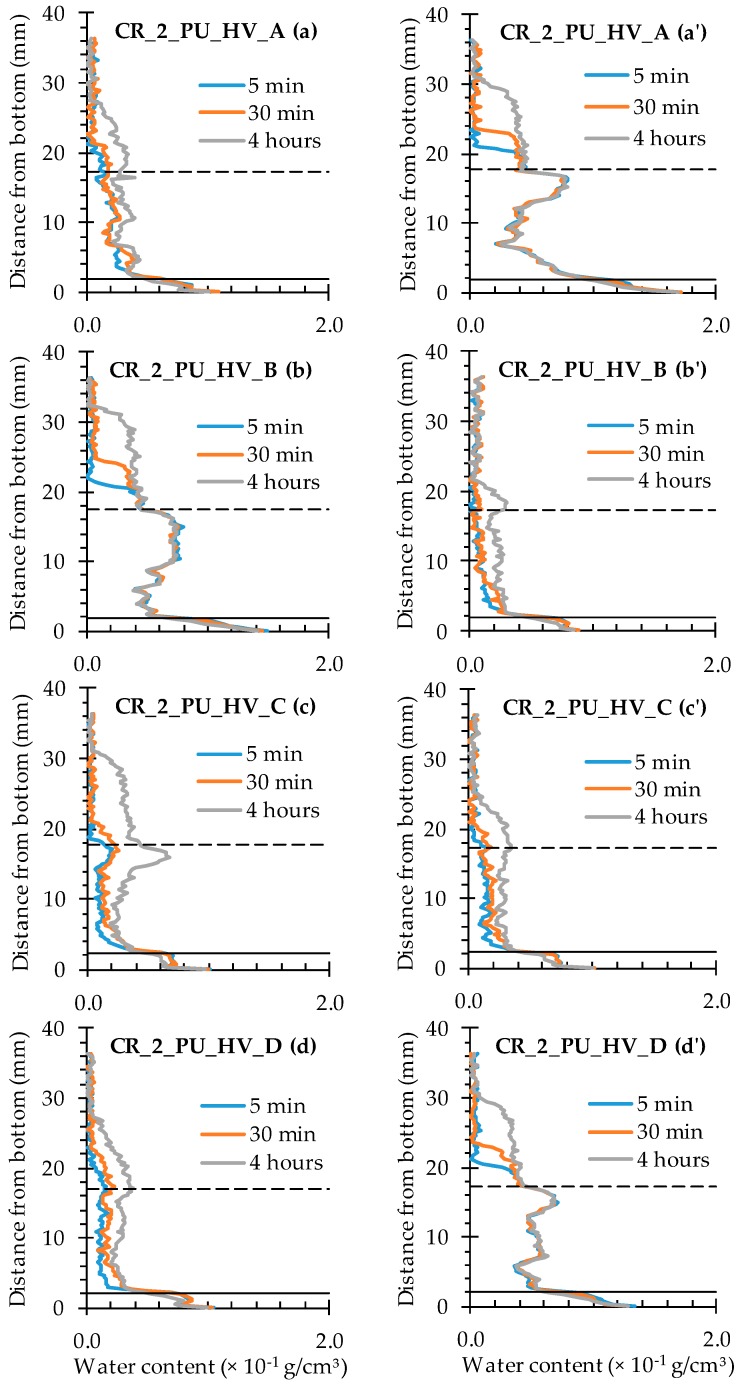
Water profiles along the crack as a function of exposure time for the neutron radiography images of the four standard mortar prisms containing two artificial cracks healed with PU_HV: (**a**) CR_2_PU_HV_A crack on the left; (**a’**) CR_2_PU_HV_A crack on the right; (**b**) CR_2_PU_HV_B crack on the left; (**b’**) CR_2_PU_HV_B crack on the right; (**c**) CR_2_PU_HV_C crack on the left; (**c’**) CR_2_PU_HV_C crack on the right; (**d**) CR_2_PU_HV_D crack on the left; and (**d’**) CR_2_PU_HV_D crack on the right.

**Figure 21:**
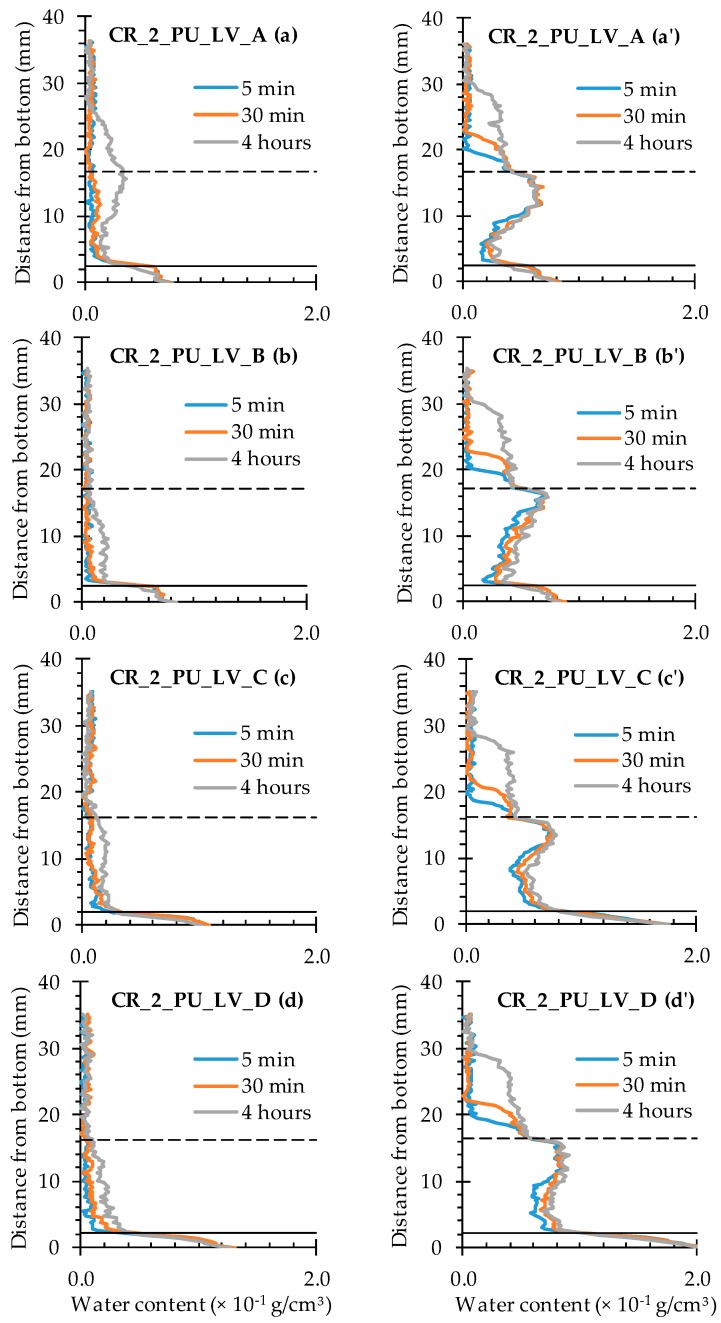
Water profiles along the crack as a function of exposure time for the neutron radiography images of the four standard mortar prisms containing two artificial cracks healed with PU_LV: (**a**) CR_2_PU_LV_A crack on the left; (**a’**) CR_2_PU_LV_A crack on the right; (**b**) CR_2_PU_LV_B crack on the left; (**b’**) CR_2_PU_LV_B crack on the right; (**c**) CR_2_PU_LV_C crack on the left; (**c’**) CR_2_PU_LV_C crack on the right; (**d**) CR_2_PU_LV_D crack on the left; and (**d’**) CR_2_PU_LV_D crack on the right.

In presence of two cracks, the amount of PU-based healing agent is seldom able to prevent increased water ingress in both of them to a sufficient extent (Figure 20 and Figure 21). With incorporation of encapsulated PU_HV (Figure 20), only sample C showed substantial healing of both cracks although the crack on the left side of the sample still showed obvious signs of water enrichment near the crack tip. The other three samples were characterized by one crack that had healed properly, while the other resembled the mere cracked condition.

For the healed cracks, an increase in water content with exposure time could still be observed in the crack region (the graph sections in between the full and dashed lines), while the water profiles of the unhealed ones did not change anymore after 5 min of exposure. It must be mentioned though that the unhealed ones still clearly differed from the cracked ones because the water content over the entire crack depth was much more variable. Usually there was a reduced water content near the bottom of the sample and water enrichment near the crack tip. The water content of the matrix above the unhealed crack tip strongly resembled those of the cracked specimens. The affected sample height was again around 30 mm after 4 h of exposure. For the healed cracks, these values ranged between 22 and 28 mm. Finally, it should be noted that the more or less adequately healed cracks could be linked systematically to the crack that was created first (the plate that was pulled out first).

For the test series containing the encapsulated low viscosity polyurethane, the water profiles clearly indicate that the available amount of healing agent could only heal one out of two cracks in all studied samples (Figure 21). The healed cracks were always those that were created first and the water content that could be achieved in them through self-healing is lower than when using the high viscosity healing agent. The matrix above the crack tip on the left side was barely affected anymore by the earlier presence of the crack. The water profiles of the unhealed cracks on the right side of the specimen again strongly resemble those of the cracked test series. The only difference is that the water content near the bottom of the samples is clearly lower than near the crack tip. Most probably, a large portion of the crack close to the exposure surface was filled with PU, but not completely. As a consequence, water was still able penetrate and reach the crack tip where almost no healing agent was present. It gives the typical water enrichment near the crack tip, which is also visible in the neutron radiography images (e.g., in Figure 8).

### 4.4. Spread Region of the Healing Agents in the Crack

#### 4.4.1. Healing of One Crack

After splitting the specimens, the crack faces of each crack were placed next to each other and a picture of the total crack surface was taken. For each of the crack faces, the total area covered by polyurethane was determined using photo editing software. The percentage of the crack face covered by polyurethane was then denoted as the surface coverage of the crack face. In this way, the spread region of the polyurethane inside the crack could be determined.

**Figure 22:**
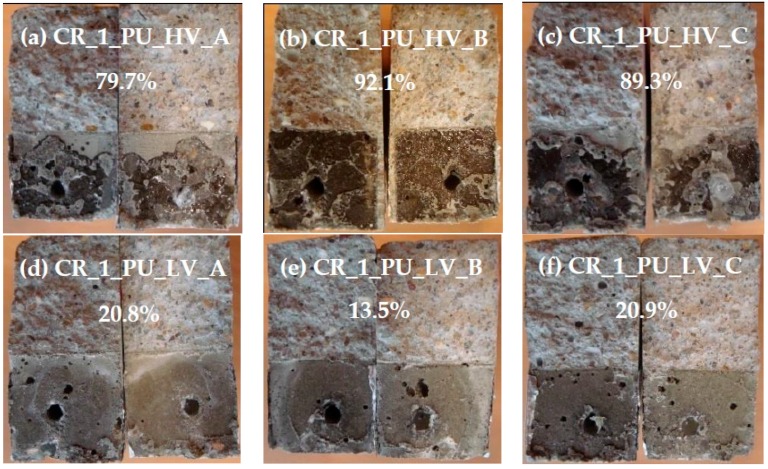
Visualization of the spread region of the healing agents in the crack for mortar prisms with one healed crack. Mean surface coverage of both crack faces is indicated for each individual specimen. (**a**) CR_1_PU_HV_A; (**b**) CR_1_PU_HV_B; (**c**) CR_1_PU_HV_C; (**d**) CR_1_PU_LV_A; (**e**) CR_1_PU_LV_B; and (**f**) CR_1_PU_LV_C.

For the specimens containing one healed crack, the pictures of the crack faces are represented in Figure 22. The mean surface coverage of both crack faces for each crack is also shown in this figure.

For the samples where the crack was healed with the high viscosity PU-based healing agent (Figure 22a–c), the surface coverage ranged from 79% to 92%. Clearly this healing agent is able to fill up most of the crack volume. This can also be noticed in the neutron radiographs (Figure 7a). The crack has a white color over the entire depth, which means that the crack was filled with healing agent to a large extent and no water could enter anymore through the crack.

The samples where the crack was healed with the low viscosity PU-based healing agent (Figure 22d–f) show a much lower surface coverage. When analyzing the crack faces, it can be seen that there is a dark color on both crack faces due to the leakage of polyurethane. However, the crack was not filled by the hardened polyurethane at those regions. A possible explanation for the dark color of the crack faces is that some minor amount of polyurethane might have been absorbed by the mortar matrix. Only at the bottom of the crack hardened polyurethane was noticed at both crack faces. This can be attributed to the very low viscosity of this PU-based healing agent. When the plates were pulled out, most of the PU leaked to the bottom of the crack and some even flowed out of the crack onto the bottom surface of the specimens. This excess PU at the bottom was then removed when the outer layer of the bottom surface was cut off to obtain a flat test face (see Section 3.2). Thus, for the specimens with the low viscosity PU-based healing agent, only 13% to 21% of the crack at the bottom was filled with healing agent. In the neutron radiography images, the incomplete filling of the crack can also be seen. In Figure 7b the inside of the crack for specimen CR_1_PU_LV_A is not completely showing the white color. There is a zone inside the crack that remains dark-colored. It further indicates that the crack is indeed not filled with the healing agent in that region.

From the analysis of the crack faces of the specimens it can be concluded that the high viscosity healing agent is able to fill up much more crack volume than the low viscosity healing agent. However, based on a mainly visual evaluation of the water uptake in the neutron radiographs (Section 4.1.2) it was concluded that the low viscosity healing agent worked more efficiently to reduce the water ingress through the crack. This might seem contradictory with the results of the spread region of the healing agents. However, it is possible that even though the low viscosity healing agent does not fill most of the crack volume, there is a good sealing of the crack near the bottom surface of the specimen in contact with water during the capillary absorption test.

#### 4.4.2. Healing of Two Cracks

**Figure 23:**
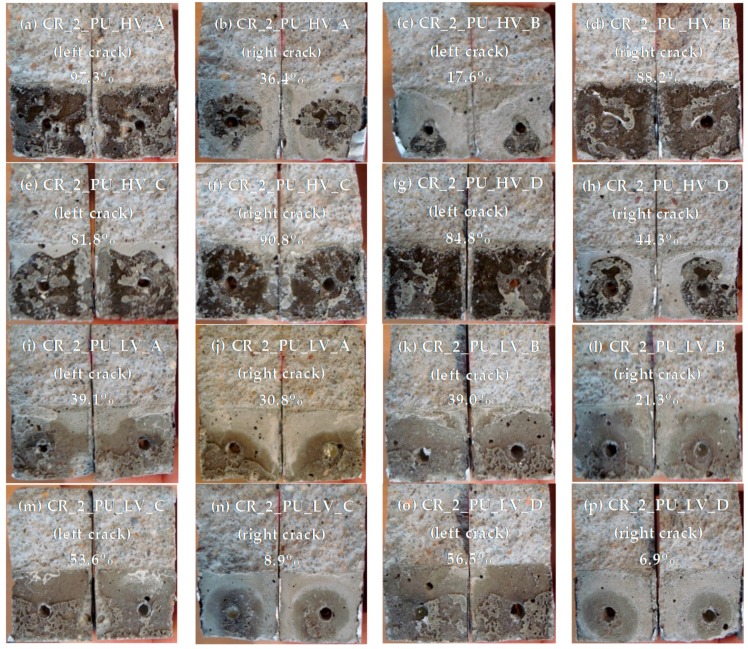
Visualization of the spread region of the healing agents in the crack for mortar prisms with two healed cracks. Mean surface coverage of both crack faces is indicated for the cracks of each individual specimen. (**a**) CR_2_PU_HV_A (left crack); (**b**) CR_2_PU_HV_A (right crack); (**c**) CR_2_PU_HV_B (left crack); (**d**) CR_2_PU_HV_B (right crack); (**e**) CR_2_PU_HV_C (left crack); (**f**) CR_2_PU_HV_C (right crack); (**g**) CR_2_PU_HV_D (left crack); (**h**) CR_2_PU_HV_D (right crack); (**i**) CR_2_PU_LV_A (left crack); (**j**) CR_2_PU_LV_A (right crack); (**k**) CR_2_PU_LV_B (left crack); (**l**) CR_2_PU_LV_B (right crack); (**m**) CR_2_PU_LV_C (left crack); (**n**) CR_2_PU_LV_C (right crack); (**o**) CR_2_PU_LV_D (left crack); and (**p**) CR_2_PU_LV_D (left crack).

The pictures of the crack faces of the samples containing two healed cracks are shown in Figure 23. For the samples where the cracks were healed with the high viscosity PU-based healing agent, it can be seen that the crack which was created first (Figure 23a,d,f,g) has the highest surface coverage (ranging from 84% to 97%). This is comparable to the values of the surface coverage that were found for the specimens with one crack healed with the same healing agent (Figure 22a–c). The surface coverage of the crack that was created last varies greatly among the different specimens. For sample CR_2_PU_HV_B only a very small amount of healing agent (surface coverage of 17.6%) was noticed in the second crack (Figure 23c). For sample CR_2_PU_HV_C the surface coverage of the second crack was 81.8% (Figure 23e). This means that the healing agent in the capsule was much more equally distributed between both cracks and the amount of healing agent was enough to fill most of the two crack volumes. The fact that both cracks are filled well can also be seen in Figure 8a where both cracks have a white color in the neutron radiographs. However, from the image of the water absorption after 4 h, it can be seen that there is a water enrichment at the crack tip of the left crack. This means that the sealing of the left crack (crack that was created last) was not perfect and due to capillary action there was some water that could penetrate to the zone of the crack that was not covered with polyurethane.

For the sample where two cracks were healed with the low viscosity PU-based healing agent, a much lower surface coverage was found for the crack that was created first (Figure 23i,k,m,o) compared to the cracks healed with the high viscosity PU-based healing agent. In accordance with the specimens containing just one healed crack, the hardened polyurethane was only found at the lower part of the crack. Again there was quite some variation in surface coverage for the crack that was created last. For the samples CR_2_PU_LV_A and CR_2_PU_LV_B the surface coverage of the second crack (Figure 23j,l) was still more than half the surface coverage of the first crack. For samples CR_2_PU_LV_C and CR_2_PU_LV_D, much less healing agent was found in the second crack (Figure 23n,p) compared to the first crack.

When comparing the visual evaluation of the water absorption in specimen CR_2_PU_LV_B (Figure 8b) to the result of the crack surface coverage (Figure 23k,l) it can be seen that no water enters through the left crack. This means that the surface coverage of 39% at the bottom of the left crack was sufficient to prevent the water from entering the crack. However, in the crack on the right side, water enters through the whole crack. Apparently, the sealing of this second crack at the bottom was not sufficient to prevent ingress of water.

### 4.5. Water Uptake Immediately after Triggering the Healing Mechanism

**Figure 24:**
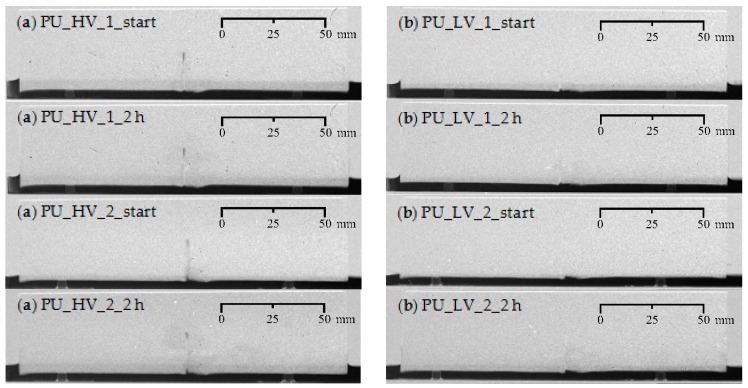
Visualization of the water uptake as a function of exposure time using neutron radiography for four mortar prisms immediately after release of the PU-based healing agent: (**a**) crack healing with PU_HV (specimens: PU_HV_1-2); and (**b**) crack healing with PU_LV (specimens: PU_LV_1-2).

In the previous experiments, the specimens with self-healing properties were only brought in contact with water after the healing agents had enough time to form a foam and harden. Usually this takes around 24 h. However, these are circumstances that are not often encountered in everyday concrete environments. Upon cracking there may already be contact with water, and the PU healing must then also work properly. Neutron radiography images of specimens containing one artificial crack, where the release of either of the two healing agents (PU_HV and PU_LV) was triggered and exposure to water began not long after the removal of the thin metal plates (D specimens PU_HV_1 and PU_LV_1: after 15 min; E specimens PU_LV_2 and PU_LV_2: after 3 h), show that this situation is not problematic at all (Figure 24). In contrast with the specimens where the PU in cracks had already hardened, these specimens clearly show a much more pronounced healing efficiency as there is almost no visible water front. This behavior can be explained as follows. Both PU precursors that have been used in this research require water to initiate the foaming reaction. When bringing the specimen in contact with water, upon release of the PU precursor, it apparently creates the optimal conditions for a profound foaming reaction. Normally, the moisture content of the cementitious matrix around the crack and the air humidity is sufficient to generate the foam. Still, with even more water around, the reaction conditions can be even more beneficial. This holds true for both the high and low viscosity healing agents.

## 5. Conclusions

Neutron radiography imaging was found to be a very effective technique to assess the healing performance of two encapsulated polyurethane-based healing agents—one with high and one with low viscosity—inside mortar upon cracking and a subsequent 4 h capillary sorption test. The water uptake as a function of time could be visualized very clearly.

From a mere visual interpretation of one representative neutron radiography image per test series, it seemed that one artificially induced crack, 300 µm in width and 20 mm deep, could be healed more efficiently by means of the low viscosity healing agent. However, comparison of the WFA/SCA ratios for all samples could not really confirm this finding as the WFA/SCA ratios varied considerably within each test series. When aiming at healing two artificially induced cracks, the same conclusions can be drawn. Evaluation of the water profiles which provided information on a more local scale, *i.e.*, a 80 mm long, thin rectangular area at height 11.3 mm from the bottom of the samples, as well as their integrals also did not indicate a substantial difference in healing performance depending on the viscosity of PU precursor. Nevertheless, the low viscosity healing agent clearly tends to heal only one of two cracks more effectively, *i.e.*, the one that was created first. Additional evaluation of water profiles taken along the cracks and the spread regions of self-healing substances onto the crack faces after the capillary sorption tests later confirmed this. Apart from that, it should be noted that the now incorporated amount of each PU precursor in the capsules was insufficient to heal one or two cracks completely.

The above-mentioned conclusions refer to the scenario in which the polyurethane foam was given enough time to harden before the mortar specimens were brought in direct contact with water. Alternatively, some specimens with one crack were immediately exposed to water after initiating capsule breakage and release of the PU precursor. Based on visual assessment of the neutron radiography images obtained during a two hour capillary sorption test it was observed that in abundant presence of water the foaming reaction was found to be much more profound and capable of preventing further water ingress almost completely in the crack zone. This was the case for both the high and the low viscosity healing agent. It implies a great potential of using encapsulated PU-based healing agents in very humid environments where the foaming reaction does not have to depend on the moisture content of the mortar and the air humidity alone.

## Figures and Tables

**Table 1:** Test series under investigation (*n* = number of specimens).

Code	Description Test Series	*n*
UN	Uncracked mortar samples	2 ^a^
CR_1	Mortar samples with one standard crack	3 ^a^
CR_2	Mortar samples with two standard cracks	3 ^a^
CR_1_PU_HV	Mortar samples with one standard crack autonomously healed with high viscosity healing agent	3 ^a^ + 2 ^b^
CR_1_PU_LV	Mortar samples with one standard crack autonomously healed with low viscosity healing agent	3 ^a^ + 2 ^b^
CR_2_PU_HV	Mortar samples with two standard cracks autonomously healed with high viscosity healing agent	4 ^a^
CR_2_PU_LV	Mortar samples with two standard cracks autonomously healed with low viscosity healing agent	4 ^a^

^a^ exposed to water after PU hardening; ^b^ exposed to water before PU hardening.

**Table 2:** Water front/sample cross-section area (WFA/SCA) ratios as calculated from the neutron radiography images after 4 h of exposure (Figure 5, Figure 6, Figure 7 and Figure 8).

Sample	WFA/SCA (%)
UN	10.8 ± 5.0
CR_1	14.1 ± 0.6
CR_1_PU_HV	16.7 ± 3.5
CR_1_PU_LV	15.7 ± 4.5
CR_2	26.9 ± 1.9
CR_2_PU_HV	25.3 ± 3.1
CR_2_PU_LV	24.0 ± 3.8

**Table 3:** Water profile integrals (×10^−1^ g/cm^2^).

Sample	Integral (×10^−1^ g/cm^2^)
UN	1.6 ± 0.5
CR_1	1.8 ± 0.1
CR_1_PU_HV	2.0 ± 0.3
CR_1_PU_LV	1.7 ± 0.4
CR_2	2.4 ± 0.3
CR_2_PU_HV	2.3 ± 0.2
CR_2_PU_LV	2.4 ± 0.2

## Abbreviations

The following abbreviations are used in this manuscript:

CR: Cracked
HV: High viscosity
LV: Low viscosity
PSI: Paul Scherrer Institute
PU: Polyurethane
UN: Uncracked
WFA/SCA: Water front/sample cross-section area ratio
